# Parasites of parasites: Herbaria as archives of hidden ecological interactions

**DOI:** 10.1098/rspb.2026.1772

**Published:** 2026-11-11

**Authors:** Jacklyn Thomas, Andrew Paul, Uma Crouch, Uwe Braun, Susumu Takamatsu, Márk Z. Németh, Levente Kiss, Ignazio Carbone, James Mitchell, Henry Van T. Cotter, Michael Bradshaw

**Affiliations:** ^1^ North Carolina State University, Center for Integrated Fungal Research, Department of Entomology and Plant Pathology, Raleigh, NC 27606, USA; ^2^ Martin Luther University, Institute of Biology, Department of Geobotany and Botanical Garden, Herbarium, Am Kirchtor 3, 06099 Halle (Saale), Germany; ^3^ Retired Professor at Graduate School of Bioresources, Mie University, 1577 Kurima–Machiya, Tsu, Mie 514-8507, Japan; ^4^ Plant Protection Institute, HUN-REN Centre for Agricultural Research, Budapest, Hungary; ^5^ Department of Plant Anatomy, Institute of Biology, ELTE Eötvös Loránd University, Budapest, Hungary; ^6^ Centre for Crop Health, University of Southern Queensland, Toowoomba, Australia; ^7^ Harvard University, Farlow Herbarium, Department of Organismic and Evolutionary Biology, Cambridge, MA 02138, USA; ^8^ University of North Carolina at Chapel Hill, University of North Carolina at Chapel Hill Herbarium, North Carolina Botanical Garden, Chapel Hill, NC 27599, USA

**Keywords:** hyperparasite, *Ampelomyces*, powdery mildew, coevolution, parasite–parasite interactions, fungal diversity, herbarium collections, host specificity, sequence reliability

## Abstract

Hyperparasitism, the infection of parasites by other parasites, is among the least explored ecological interaction types. *Ampelomyces*, intracellular fungi that infect the plant diseases causing powdery mildews, form a naturally occurring parasite-on-parasite system that is both widespread and poorly understood. This tripartite association among plants, pathogens and hyperparasites provides a powerful model for studying multi-trophic interactions and the evolution of host specialization. Using DNA from herbarium specimens collected across 28 countries over the past 150 years as well as those available in NCBI GenBank, we revealed previously hidden ecological and evolutionary patterns in the *Ampelomyces*–powdery mildew system. Historical collections allowed us to detect hyperparasites that cannot be easily observed, uncovering multiple novel lineages and global patterns of host association. A global reassessment of all publicly available sequences showed that 13% of entries labelled as *Ampelomyces* are misidentified, revealing a major issue with uncurated, public databases. To resolve this, we developed a curated, voucher-linked phylogenetic framework in T-BAS to serve as a reference for future ecological, genomic and biocontrol studies. Together, our results demonstrate that herbaria function as large-scale ecological observatories, capable of revealing cryptic interactions across continents and providing a strengthened framework for future ecological and evolutionary research on hyperparasitism.

## 1. Introduction

Fungi that parasitize other fungi are known as mycoparasites [1–3]. *Ampelomyces* species, including *Ampelomyces quisqualis*, represent one of the few classic examples of mycoparasites that specifically target obligate biotrophic fungal parasites; therefore, they are also known as hyperparasites [4–10]. The obligate biotrophic mycohosts of *Ampelomyces*, the powdery mildews (Erysiphaceae), include over 900 species belonging to 18 genera [11–16]. In addition to being plant parasites, the powdery mildews are also known as common plant pathogens, that is, disease-causing agents, as they cause disease of over 10 000 angiosperm species, including important crops [11,17]. As intracellular antagonists of powdery mildews, *Ampelomyces* species have evolved to exploit a highly specialized ecological niche that poses unique selective pressures. This unusual trophic interaction—where a mycoparasite parasitizes an obligate biotroph—provides a powerful model for exploring the evolutionary dynamics of hyperparasitism, fungal host specificity and co-evolution within the fungal kingdom.

*Ampelomyces* is a globally distributed fungal genus and has been reported on many powdery mildew species on a wide range of host plants, including economically important crops such as wheat, barley, grapevine and many vegetable and fruit species [7,18–27]. Although host–pathogen patterns between *Ampelomyces* strains and powdery mildew species have been observed [8,24,26], this relationship remains difficult to interpret owing to the widespread ability of *Ampelomyces* strains to cross-infect multiple powdery mildew mycohosts [8,18,22,23,25,26]. However, strain-level variation in pathogenicity and virulence has also been documented, with outcomes depending on the specific *Ampelomyces*–powdery mildew pairing [28]. For example, *Ampelomyces* germination and hyphal growth can be influenced by temperature and host-derived cues, with some strains being able to germinate on hosts that are different from the original host they were collected on [23,28]. The presence of significant genetic and phenotypic variation among *Ampelomyces* strains [28–30] implies that distinct ecological adaptations have evolved, potentially shaping mycohost–mycoparasite interactions. One hypothesis for observed genotype–host associations is temporal isolation, as different powdery mildew hosts undergo epidemic cycles at different times of the year, possibly restricting opportunities for cross-infection [8].

Despite these insights, major gaps remain in our understanding of the global diversity and evolutionary dynamics of *Ampelomyces*. Most studies have focused on strains from Europe [4,8,21,25–28], Asia [22–24,29] and Australia [18], with North America remaining under-represented. As a result, our current view of *Ampelomyces* diversity is limited by narrow geographical sampling, hampering efforts to resolve evolutionary relationships and assess the full extent of ecological and genetic variation within this genus. Broader, global sampling—including from diverse powdery mildew hosts—will be critical to disentangle host specificity, geographical structure and co-evolution.

Herbaria offer unparalleled access to historical and geographically diverse collections of powdery mildews, making them powerful resources for uncovering hyperparasite–host interactions that are otherwise difficult to observe in natural settings. Using the extensive diversity of powdery mildew specimens housed in the Larry F. Grand Mycological Herbarium (NCSLG) at North Carolina State University [31], our primary objectives were (i) to characterize the global diversity of *Ampelomyces*, with an emphasis on North America—an under-represented regionand (ii) to assess whether *Ampelomyces* lineages show associations with particular powdery mildew hosts. Previous studies have documented substantial genetic variation in both the internal transcribed spacer (ITS) region of the ribosomal DNA (rDNA) and actin (*ACT*) regions of *Ampelomyces* [8,22,25,30,32], and during the development of species-specific primers, we also encountered widespread misidentification among GenBank accessions, revealing that the issues presented by Bidartondo *et al.* [33] are still prevalent. To overcome these limitations, we incorporated our validated sequences into T-BAS, a curated and open-access platform for phylogenetic and population genetic analysis, providing a standardized reference framework for the accurate identification of *Ampelomyces* strains.

## 2. Material and methods

### (a) Specimen acquisition

DNA extraction for the powdery mildew specimens evaluated in this study was initially performed during the production of various manuscripts focusing on developing a North American Monograph of the powdery mildews [12,31,34–40]. All specimens evaluated are in the NCSLG or the Farlow Herbarium (FH) at Harvard University. In total, 609 herbarium specimens were screened for the presence of *Ampelomyces*. Many of the specimens at NCSLG were gifted to the herbarium from Martin Luther University Herbarium (HAL) and National Museum of Nature and Science, Tokyo (TNS) and consisted of collections from throughout the world.

### (b) Sequencing

DNA extractions were performed using the Chelex method [41,42]. Owing to their prevalent usage in previous work evaluating *Ampelomyces,* the ITS and *ACT* regions were chosen as targets for polymerase chain reaction (PCR) [8,22,25,30,32]. Specific primers were designed, and PCR was run as described by Bradshaw *et al.* [34] to amplify an approximately 300 bp amplicon of both regions. For the targeted ITS region, which spans part of ITS1, 5.8S, and part of ITS2, the primer pair AMP_ITS_F (GGTTTCCGTAGGTGAACCTG)/AMP_ITS_R (CACTGGCTGCCAATTGCTTTG) was used at an annealing temperature of 60°C. For the *ACT* region, the primer pair AMP_ACT_F (CGATCTGACTGACTACCTCA)/AMP_ACT_R (TTCCACCGCTCTCGAGACCC) was used at an annealing temperature of 60°C. For identification of the powdery mildews, the primer pairs PM10 [43]/PM2 [44] were used to target the ITS rDNA region at an annealing temperature of 58°C. For a select few powdery mildew specimens, sequencing of the ITS region was unsuccessful, and instead the rDNA intergenic spacer (IGS) region was sequenced using the primer pair IGS-12a/NS1R [45]. DNA amplicons were sent to Genewiz (South Plainfield, NJ) to be purified and directly sequenced in the forward and reverse directions using the primer pairs above.

### (c) Phylogeny

To supplement the herbarium specimen analyses, all data from GenBank, labelled as ‘*Ampelomyces*’, were downloaded and the metadata were mined (supplementary material, table S1). Using the combined Herbarium and GenBank dataset, trees were generated from the concatenated sequences of ITS and *ACT* regions. *Cippumomyces mortalis* was selected as an outgroup taxon owing to it being divergent from *Ampelomyces* and all fungi potentially misidentified as *Ampelomyces*, and because it had ITS and *ACT* sequences available on GenBank. In the phylogenetic analysis, specimens with only *ACT* sequences and no ITS data were excluded. Sequences were aligned and edited using Geneious Prime 2025.0.3. The large tree (figure 1) included all the data from GenBank labelled as *Ampelomyces*, including some of the specimens sequenced for this study (673 strains in total). Many of the sequences from GenBank were misidentified and came from several disparate genera. As such, there were many gaps in the ITS alignment that were manually removed. The identifications were confirmed by the BLAST search function coupled with limiting the results to ‘sequences from type material’. A GTR + G + I evolutionary model was used for phylogenetic analyses. This model has been shown to perform well across a wide range of datasets and generally yields phylogenetic estimates comparable to those obtained using formally selected models [46]. The phylogeny was inferred using Bayesian analysis of the combined loci using a Yule tree prior [47] and a strict molecular clock, in the programme BEAST v.1.10.4 [48]. A single Markov chain Monte Carlo chain of 10^7^ steps was run, sampling every 1000th generation, with a burn-in of 25%. Posterior probabilities were calculated from the remaining 7500 sampled trees. A maximum clade credibility tree was produced using TreeAnnotater v.1.10.4 (part of the BEAST package). Stationarity was confirmed by running the analysis multiple times, which revealed convergence between runs. Additional phylogenies were inferred using maximum likelihood analysis via RAxML [49] using the GTRGAMMA evolutionary model. Bootstrap supports and trees were obtained by running a rapid bootstrap analysis of 1000 replicates [50]. The resulting trees were visualized using FigTree v.1.3.1 [51]. The best tree, alignments and specimen metadata (e.g. host, locality) were uploaded to T-BAS [52,53] to enable real-time phylogeny-based placement of unknown *Ampelomyces quisqualis* strains for any of the loci in the reference tree.

**Figure 1.**
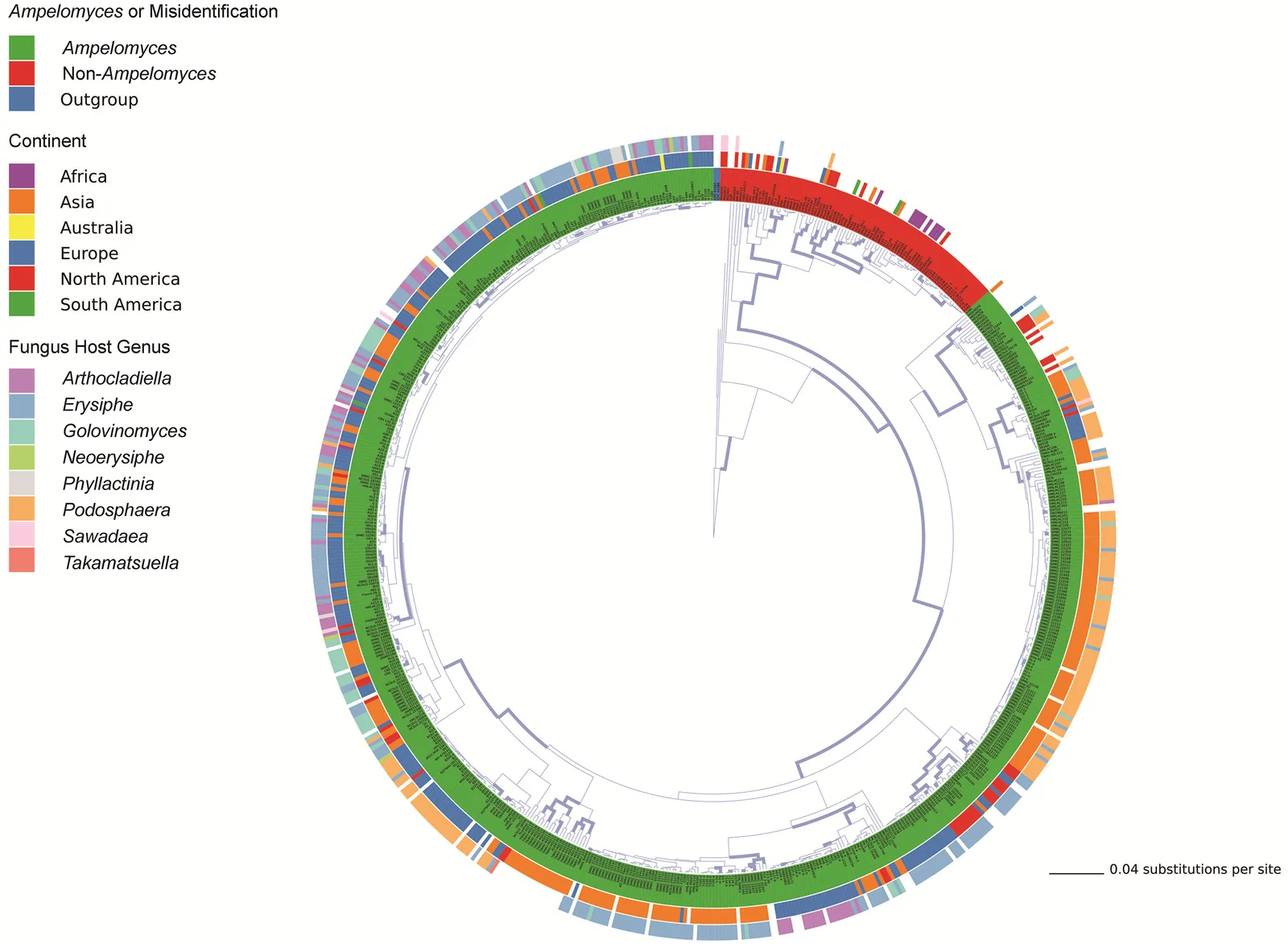
Bayesian maximum clade credibility tree of all strains labelled as ‘*Ampelomyces*’ on GenBank for a total of 673 included strains. Branches with thick lines indicate posterior probabilities greater than 0.7. All strains contain sequences for at least the ITS barcode region, with a portion of strains also including the *ACT* coding region when available. Colourized rings around the tree are used to show different strain attributes. The inner ring displays the identification of the strains as either part of the *Ampelomyces* genus or as misidentifications on GenBank. The middle ring displays the continent where the strain was collected. The outer ring displays the powdery mildew host genus associated with each strain. Specimens from ‘NCSLG’ or ‘FH’ were sequenced for the current study.

A reference phylogeny was constructed (T-BAS accession 7WPX5ZIQ) [54], using confirmed *Ampelomyces* strains for which both ITS and *ACT* sequences were available, supplemented with a subset of representative strains from Goldspink *et al.* [18]. The 47 *Ampelomyces* strains included in this tree were assigned to molecular operational taxonomic units (MOTUs) following the framework established by Goldspink *et al.* [18]. This reference tree functions as a ‘living phylogeny’ within T-BAS, enabling researchers to upload their own sequences and automatically infer their placement within the appropriate MOTU.

### (d) Haplotype network

Network inference from sequences downloaded from GenBank and those generated in this study was performed using the statistical parsimony method of TCS 1.21 [55], as implemented in DeCIFR [56]. In TCS, sequences were collapsed into haplotypes with gaps treated as a fifth state and a connection limit of 95. Nodes were coloured based on fungal host genus, and node size was proportional to haplotype frequency. Eight of the *Ampelomyces* specimens originally included in the network grouped outside of the main network and were excluded from the final analysis. These specimens contained large insertions or deletions in the ITS region that resulted in poor placement with the rest of the dataset that probably led to their disconnection in the network. Their exclusion did not impact overall patterns of connectivity.

### (e) Morphology

Morphological observations were conducted on *Ampelomyces*-infected specimens that were detected via PCR using adhesive tape and direct mounting. Clear tape was applied to infected colonies and transferred to microscope slides with a drop of distilled water. In some cases, pycnidia were mounted directly using a sterile needle. Images were captured using a Nikon Eclipse Ti2 compound microscope and a Nikon SMZ1270 dissecting microscope, both equipped with a Nikon DS-Ri2 camera. Measurements were performed using Nikon NIS-Elements software.

## 3. Results

### (a) *Ampelomyces* prevalence

In the course of the study, we evaluated 609 herbarium specimens to determine whether *Ampelomyces* was present. These samples originated from 28 countries (Argentina, Brazil, Canada, China, Costa Rica, Croatia, France, Germany, Hungary, India, Iran, Japan, Kazakhstan, Lithuania, Mexico, Montenegro, Morocco, the Netherlands, Peru, Portugal, Russia, South Korea, Spain (the Canary Islands), Sweden, Switzerland, Tanzania, Ukraine and the United States). Overall, *Ampelomyces* was detected by PCR in 40 of the 609 specimens evaluated (supplementary material, table S2), yielding an infection rate of 6.6%. The presence of *Ampelomyces* was confirmed by microscopy on all 40 specimens that were identified via PCR. Among the hosts, *Sawadaea* exhibited the highest incidence of infection at 14.3%, followed by *Takamatsuella* (12.5%) and *Golovinomyces* (12.3%). Moderate incidence was observed in *Erysiphe* (7.2%), whereas *Podosphaera* (3.2%) and *Phyllactinia* (2.7%) showed low prevalence (supplementary material, table S2). No *Ampelomyces* infections were detected in specimens of *Blumeria*, *Cystotheca* or *Pleochaeta* with the PCR approach. One *Blumeria* specimen (NCSLG25146) was observed morphologically but sequencing failed. All sequence data were deposited in GenBank (supplementary material, table S3).

### (b) Phylogeny and data mining

The metadata for the *Ampelomyces* sequences from GenBank was mined for country and host information (supplementary material, table S1). In total 671 fungal sequences that were labelled as ‘*Ampelomyces*’ were downloaded from GenBank, along with two designated outgroup samples. We placed all these sequences into a large phylogenetic tree (figure 1).

In the Bayesian analysis, 88 of the isolates fell outside of the main, moderately supported (greater than 0.7 posterior value) *Ampelomyces* clade and were thus probably incorrectly labelled as *Ampelomyces* when submitted to GenBank. Select specimens that placed within the non-*Ampelomyces* clade were confirmed to belong to other genera via BLAST search while limiting the results to ‘sequences from type material’. For example, strain F109 was labelled in GenBank as ‘*Ampelomyces* sp.’, but its ITS sequence had 100% similarity to type sequences of the genus *Epicoccum*. Similarly, strain Po58 was a 98% match with type sequences of the genus *Epicoccum* and strain MosID22 was a 93% match with type sequences of the genus *Ramularia*. All of these fell outside of the *Ampelomyces* clade and were labelled in the present study as non-*Ampelomyces.* Even some specimens deposited in culture collections were misidentified as *Ampelomyces*. For example, ATCC 38616 was identical to type sequences of *Didymella glomerata* and ATCC 201059 showed 98.5% similarity with type material from *Stagonosporopsis lupini.*

It should be noted that in the maximum likelihood analysis there was minimal support for the *Ampelomyces* and non-*Ampelomyces* clades (supplementary material, figure S1). However, these relationships were inconsistent with sequence identities obtained through BLAST comparisons with type material. For example, Eg8 (GQ244508) grouped inside the *Ampelomyces* clade in the maximum likelihood analysis, yet inside the non-*Ampelomyces* clade in the Bayesian analysis. When this strain was entered into a BLAST search, it had high identity (more than 99% homology) with isolates in the genus *Pichia*. Another example is AQZ (JF441270) that falls in the *Ampelomyces* clade in the maximum likelihood tree but appears most similar to *Aureobasidium* species (less than 99% homology) based on a BLAST search. It should be noted that strain AMN (PV268082), as well as others originating from India (many with the prefix: TNAU), are shown by BLAST search to be distantly related to all other fungi, yet the closest matches are other *Ampelomyces* accessions (approx. 85% homology). As such, their identification as *Ampelomyces* is unclear and requires future investigation, such as inoculation studies.

Of the 671 downloaded *Ampelomyces* sequences from GenBank, we were able to place 583 (approx. 87%) into the *Ampelomyces* clades, whereas approximately 13% probably represented misidentifications. It should be noted that there is relatively low support for the overall *Ampelomyces* clade (posterior probability = 0.77; figure 1). The phylogenetic analysis was limited by the fact that it was based primarily on ITS data, but nonetheless offers a useful framework to assess whether their isolates are genuinely *Ampelomyces*. Future studies incorporating additional genetic loci will be essential to resolving relationships within the genus and improving taxonomic resolution.

The 40 sequences generated from the herbarium specimens in the current study, as well as the confirmed specimens from GenBank, were found throughout the world on a range of powdery mildew hosts (supplementary material, table S1). From the specimens on GenBank, *Erysiphe* were the most reported host of *Ampelomyces* (43.9% of all reports; supplementary material, table S2). However, this frequency reflects sampling effort rather than prevalence, because *Erysiphe* is the most speciose and commonly collected genus of powdery mildews, containing roughly half of all species [13]. We therefore interpret the GenBank host distribution as a reflection of collection and sequencing bias, not relative infection rates.

The reference tree was generated from 47 confirmed *Ampelomyces* specimens and two outgroup specimens with both ITS and *ACT* data, and is available in T-BAS for viewing and phylogenetic placement (reference tree accession 7WPX5ZIQ; figure 2 ) [54]. This tree shows all nine MOTUs revealed based on an ITS analysis by Goldspink *et al.* [18]. Our analysis also revealed additional MOTUs different from those previously reported. This is unsurprising considering the low amount of data that were available from North America before our study.

**Figure 2.**
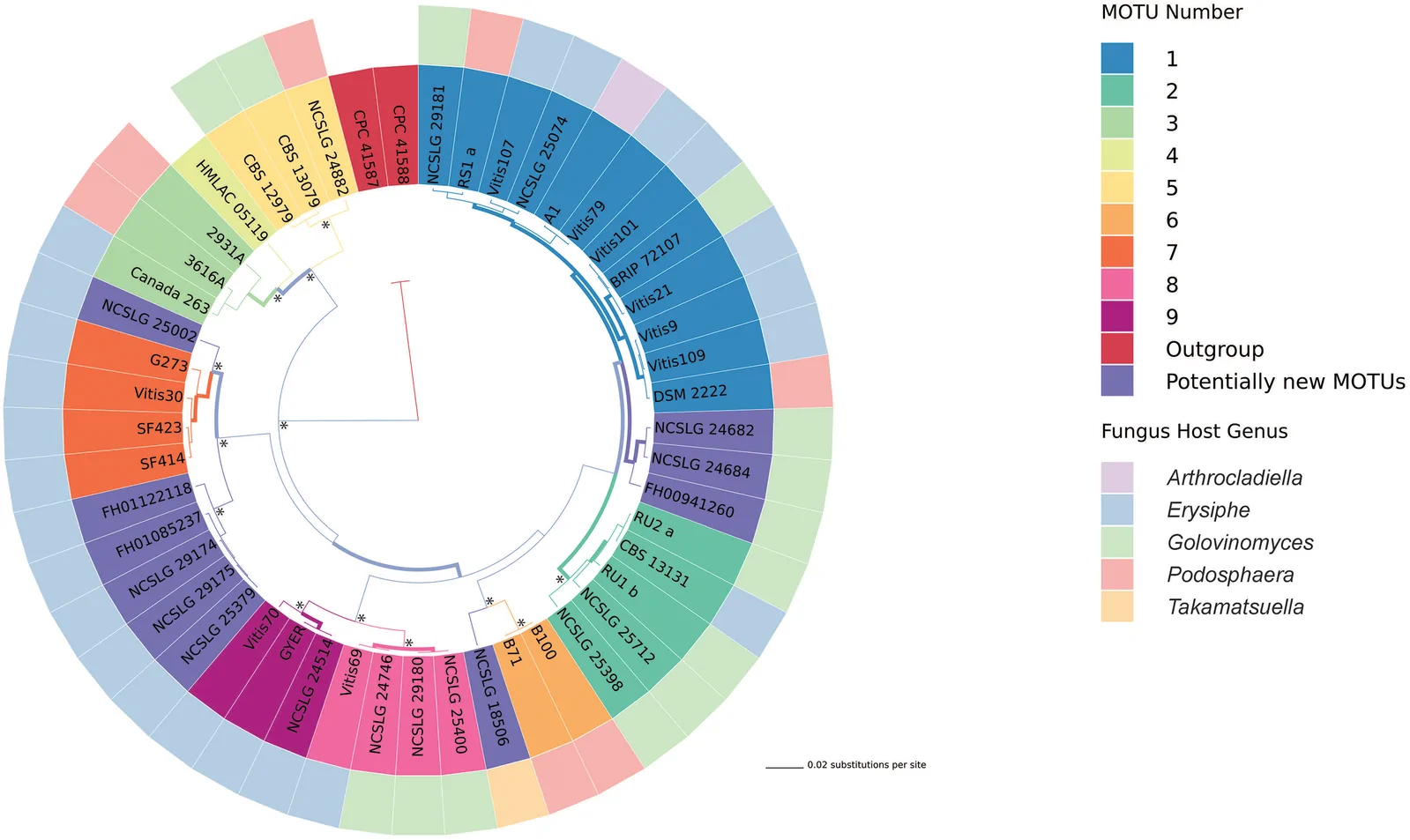
Bayesian maximum clade credibility tree of the 47 *Ampelomyces quisqualis* specimensthat belong to the T-BAS reference tree. Branches with thick lines indicate posterior probabilities greater than 0.7. Bootstrap values above 50 from the maximum likelihood analysis are noted with an asterisk to indicate where there was support for the same clades as in the Bayesian analysis. At least one representative sample from all previously established MOTUs was included in the tree that had both ITS and *ACT* regions sequenced. Specimens from ‘NCSLG’ or ‘FH’ were sequenced for the current study. Colourized rings around the tree are used to show different strain attributes. The inner ring displays the identification of the strains as belonging to a distinct MOTU as defined by Goldspink *et al.* [18]. The outer ring displays the fungal host genus that the strain was found to be associated with.

### (c) Haplotype network

To investigate patterns of genetic diversity and host association among *Ampelomyces* isolates, a haplotype network was constructed for the concatenated ITS and *ACT* data (figure 3). The network revealed extensive haplotype diversity and structured relationships among isolates. Several well-connected clusters were observed revealing powdery mildew genus-*Ampelomyces* relationships. For example, strains associated with *Arthocladiella* tended to group together, as did those from *Podosphaera*. *Erysiphe*-associated strains were distributed broadly across the network, often occurring in shared haplotypes with other powdery mildew genera. Some clusters appear more diverse in terms of powdery mildew host genera than others, indicating that some genetic clusters are associated with wider ranges of fungal hosts than others.

**Figure 3.**
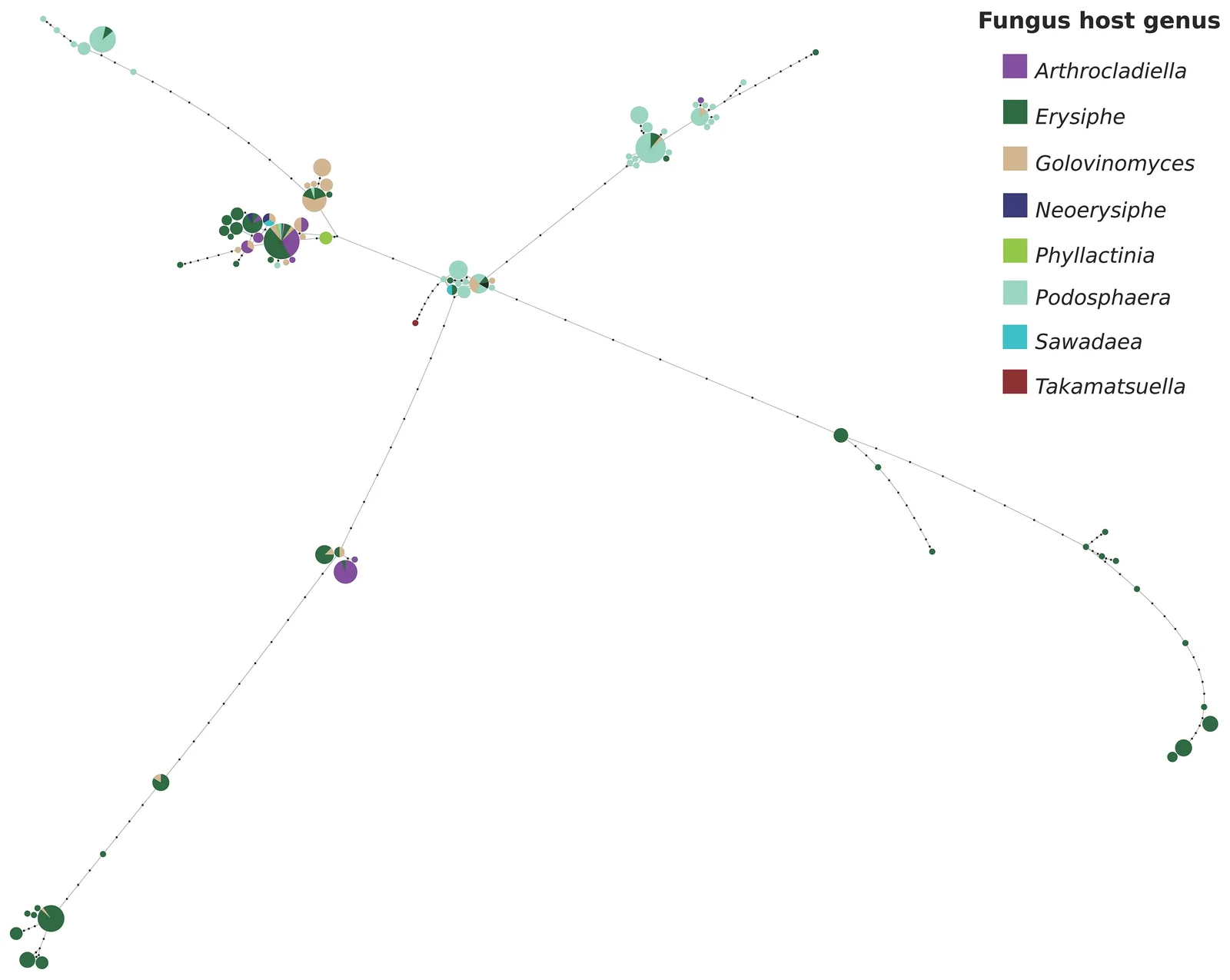
Statistical parsimony haplotype network visualization of *Ampelomyces quisqualis* strains analysed in the present study, with nodes coloured as a function of fungal host genus. Sequences evaluated included newly generated data from herbarium specimens as well as those from GenBank. Node size is proportional to haplotype frequency. Non-*Ampelomyces* misidentifications noted from figure 1 were excluded from the network, with the disconnected outgroup also excluded.

### (d) Morphology

We proceeded to morphologically confirm the presence of *Ampelomyces* on all 40 herbarium collection samples that were positively identified as *Ampelomyces* from the sequencing data. A representative figure illustrating the morphologies observed across various powdery mildew and plant genera is shown in figure 4.

**Figure 4.**
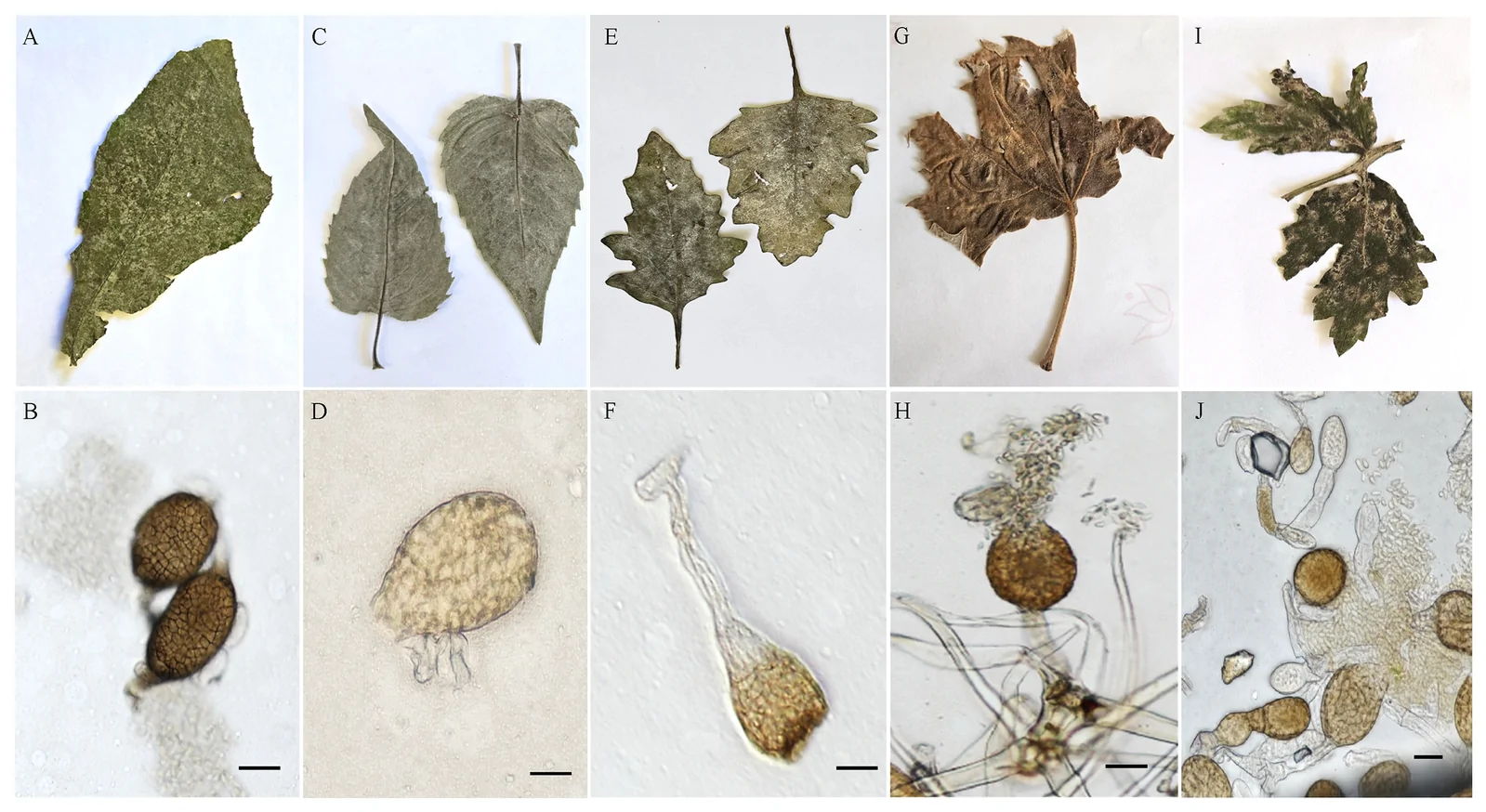
*Ampelomyces* infecting multiple powdery mildew genera. (A and B) *Ampelomyces* parasitizing *Podosphaera xanthii* (scale bar, 10 μm) on *Cucurbita pepo*. (C and D) *Ampelomyces* parasitizing *Golovinomyces monardae* (scale bar, 10 μm) *on Monarda clinopodia*. (E and F) *Ampelomyces* parasitizing *Erysiphe aquilegiae* (scale bar, 10 μm) on *Nicandra physalodes*. (G and H) *Ampelomyces* parasitizing *Erysiphe platani* (scale bar, 10 μm) on *Platanus* sp. (I and J) *Ampelomyces* parasitizing *Golovinomyces hydrophyllacearum* (scale bar, 20 μm) on *Hydrophyllum virginianum*.

## 4. Discussion

This study provides a global, herbarium-based perspective on the evolutionary and ecological structure of a tripartite interaction among host plants, obligate plant pathogens and their intracellular hyperparasites, *Ampelomyces*. By integrating phylogenetic inference, haplotype network analyses, morphology and extensive voucher-linked metadata, we reveal that *Ampelomyces* diversity is far greater and more structured than previously noted, with clear implications for host specialization and parasite–hyperparasite coevolution. Our analyses identify multiple novel lineages, demonstrate a continuum of host preference across *Ampelomyces* genotypes from general to more specialized, and expose a high rate of global misidentification in public databases. Importantly, these patterns suggest that interactions between powdery mildews and *Ampelomyces* are not random but instead reflect lineage-specific compatibility and potentially temporal and/or ecological partitioning among hosts. Herbarium specimens provide a unique resource for evaluating these complex ecological relationships. They capture a snapshot of the naturally occurring interactions as they existed at a point in the past and allow for the consideration of environments across broad spatial and temporal scales, while avoiding the time and expense of traditional sampling over the same area and without the artificial constraints or selection biases imposed by controlled laboratory or greenhouse experiments. As a result, herbarium-based studies provide a rare window into realized host–pathogen–hyperparasite associations, enabling robust inference of ecological and evolutionary dynamics that cannot be readily reconstructed from experimental systems alone. Together, these features position the *Ampelomyces*–powdery mildew system as a powerful model for understanding parasite-of-a-parasite dynamics and the evolutionary processes shaping complex, multi-trophic associations.

Variation in *Ampelomyces* prevalence across powdery mildew genera offers important insights into host–parasite interactions and the ecological or evolutionary factors shaping infection dynamics. Overall, the mycoparasitism rate observed in our study was 6.6%. This is substantially lower than the 32.75% and 36% mycoparasitism rates reported from Serbia and Hungary, respectively, both of which were based on morphological examination of large numbers of field samples [21,57]. The discrepancy may be owing to sampling bias and geographical differences in sampling, as our study took a worldwide approach. Additionally, methodological factors may have contributed: our DNA extractions were performed on scraped powdery mildew material without specifically targeting *Ampelomyces*, raising the possibility that some infections were present but not detected. In this work, the highest prevalence was observed in *Sawadaea* (14.3%), *Takamatsuella* (12.5%) and *Golovinomyces* (12.3%; supplementary material, table S2). Although these values may partially reflect small sample sizes, they may also suggest increased ecological compatibility between these genera and *Ampelomyces*.

A central question in *Ampelomyces* research is to what degree do these mycoparasites exhibit host specificity versus acting as generalists, capable of infecting diverse powdery mildew species. Although certain *Ampelomyces* genotype/MOTU–mycohost associations have been documented [8,24,26], other studies indicated that *Ampelomyces* genotypes belonging to distinct MOTUs can readily parasitize powdery mildew species other than their mycohosts of origin in laboratory tests [18,22,23,58–60] and field experiments [8]. Also, *Ampelomyces* strains representing diverse MOTUs were trapped from the air with potted grapevine plants infected with powdery mildew (*Erysiphe necator*) in a small-scale experiment [25]. Recently, this relationship has been termed ‘structural specificity’ to describe the *Ampelomyces*-powdery mildew system [30]. One proposed explanation for this apparent paradox is temporal isolation: powdery mildew hosts often experience outbreaks at different times of year, potentially limiting opportunities for cross-infection [8]. Another explanation could be that some *Ampelomyces* lineages are more associated with certain powdery mildew species or genera, whereas others are generalist mycoparasites of any powdery mildews [26]. Our current study indicates patterns of host association, as seen in both the phylogenetic tree and haplotype network (figures 1 and 3). The haplotype network, in particular, reveals substantial genetic diversity and a mixture of generalist and host-associated lineages (figure 3). For example, clusters linked to *Podosphaera* and *Arthocladiella* suggest possible specialization, whereas the broader distribution of *Erysiphe*-associated strains, falling in multiple lineages, indicates generalist behaviour. Similar patterns of seeming adaptation to a fungal host, but still imperfect host specificity, were observed in previous haplotype network analysis by Liyanage *et al.* [29]. These patterns suggest that lineage-specific genetic or ecological factors may influence host selection. Assessment of co-phylogeny between *Ampelomyces* and its associated powdery mildew hosts would be a valuable next step in the study of this interaction. Evaluating the shared evolutionary history of these organisms would require additional sampling and sequencing of additional regions, including full genomes, and would shed more light on the strength, prevalence and potential evolutionary origin of host associations in *Ampelomyces*. It is possible there are complex, unseen processes influencing the host range, such as with *Fusarium oxysporum* f. sp. *fragariae*, where a horizontally transferred pathogenicity chromosome (chrY-frag) governs host range, enabling genetically distinct strains to infect the same host [61]. Similar or unknown mechanisms may underlie host associations in *Ampelomyces*, though broader geographical and taxonomic sampling will be required to test this hypothesis.

Our phylogenetic analysis showed that the confirmed *Ampelomyces* isolates are both globally distributed and genetically diverse, with clear phylogenetic patterns (figures 1–3). Several specimens sequenced for the current study in our phylogeny fall outside currently known clades [18] and may represent novel MOTUs (figure 2). Some are associated with specific hosts, such as *Takamatsuella*, whereas others show no consistent host or geographical pattern (figures 1 and 2). For example, those from Asia, North America and Europe seem to loosely group together in the phylogeny (figure 1). Notably, we observed *Ampelomyces* pycnidia on *Blumeria bulbigera* (NCSLG 25146) based on our morphological analyses. Although *Blumeria* species have been reported as occasional hosts of *Ampelomyces* in Hungary [20], Korea and Czechia [26], infection rates were exceptionally low in a field study [21], whereas *Ampelomyces* was not identified at all in another field study that included powdery mildew samples from 17 species of the Poaceae [57]. No sequence data were recovered from the specimen examined in this work owing to failed amplification, and no confirmed *Ampelomyces* sequences from *Blumeria* hosts were available on GenBank. Given the infrequency of this host association and the absence of molecular data, *Ampelomyces* infecting *Blumeria* spp. may represent a novel MOTU. As previously mentioned, sequence divergence among *Ampelomyces* isolates can be significant, particularly in the ITS region [22,30,32]. The high divergence of the ITS region among *Ampelomyces* could have contributed to PCR failure, in this case owing to primer mismatches. This could be another reason for our low incidence reported (supplementary material, table S2) compared with previous research [21,57]. Additionally, because this is among the first studies to assess *Ampelomyces* using herbarium specimens, it is possible that some lineages cannot be cultured, which may account for the greater diversity observed here. These distinctions underscore the importance of expanded geographical sampling on a wider range of hosts to better understand *Ampelomyces* diversity.

One of the main findings from the current study was that approximately 13% of GenBank records labelled as *Ampelomyces* are misidentified. These misidentifications distort estimates of host specificity and phylogenetic relationships, and they illustrate the need for specimens linked to vouchers or culture collections. One point to note is that even strains deposited in reputable culture collections were found to be misidentified, and the purchase of these cultures can be expensive. For example, ATCC38616 (*Didymella* sp.) and ATCC201059 (*Stagonosporopsis* sp.) are listed for sale for $5000 USD as of July 2025. However, it remains possible that these strains were originally *Ampelomyces* but became contaminated only after being acquired by the laboratories that subsequently analysed and sequenced them. Nonetheless, misidentification of diverse fungal strains with *Ampelomyces* has already been highlighted by Sullivan & White [62], Kiss *et al.* [7] and Kiss [6]. These misidentifications can also have large taxonomic implications. For example, recently *Ampelomyces quercinus* was transferred to the genus *Phoma* [63] and later *Nothophoma* [64] and has since been reported as a pathogen of various woody plants circumglobally (e.g. [65–69]). Based on comparisons of descriptions (e.g. [63,70,71]), these shifts seem to have been proposed on the basis of a misidentified *Ampelomyces* culture at CBS (Centraalbureau voor Schimmelcultures, now part of Westerdijk Fungal Biodiversity Institute) (CBS 633.92), which is also available at ATCC (ATCC 36786).

Collectively, these results highlight the evolutionary and ecological complexity of the *Ampelomyces*–powdery mildew system. As a mycoparasite of obligate plant pathogens, *Ampelomyces* represents a compelling example of a parasite-of-a-parasite system. The observed genetic structuring and genotype–host associations suggest that evolutionary processes such as phylogeny, host expansions and temporal isolation may shape patterns of infection. This system offers a powerful model for investigating host specialization, adaptation and the dynamics of tripartite interactions among plants, pathogens and hyperparasites. Expanding sampling across diverse hosts and geographical regions will deepen our understanding of the evolutionary pressures driving these relationships and further establish *Ampelomyces* as a valuable system for studying coevolution and parasitic complexity.

## Supplementary Material



## Data Availability

All data generated in this study are available in GenBank [72], with accession numbers provided in supplementary Table S3. All specimen data are available in supplementary Table S3 and the Mycology Collections Portal [73]. Supplementary material is available online [74].
