# Supplementary material for: Parasites of parasites: Herbaria as archives of hidden ecological interactions
Source: Proc Biol Sci. 2026 Nov 11;293(2082):20261772. doi: 10.1098/rspb.2026.1772 (PMC13751125; doi:10.1098/rspb.2026.1772)
Supplement: Supplementary file 1 [file rspb.2026.1772.zip › rspb.2026.1772/rspb20261772_si_004.pdf]

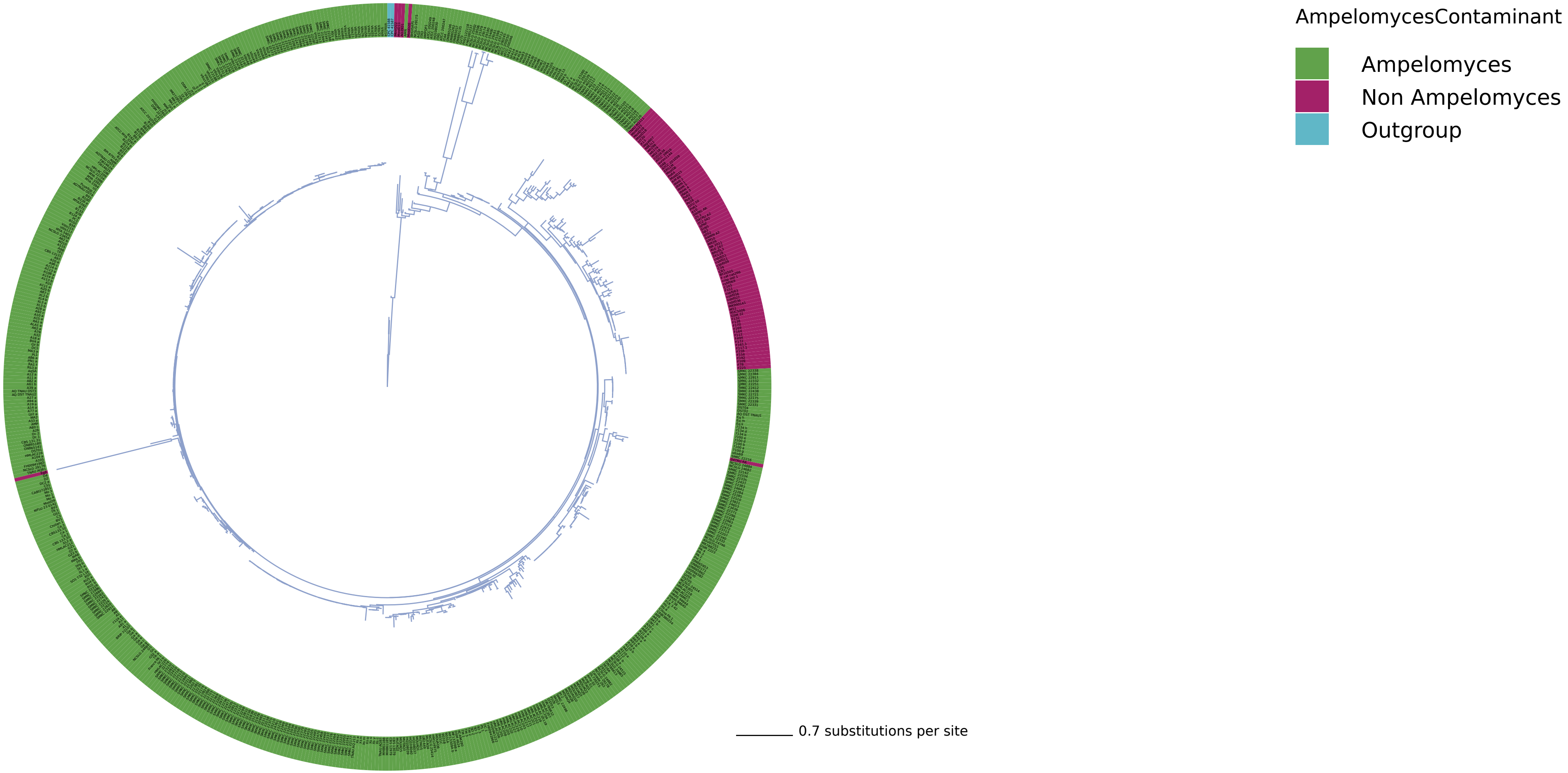
AmpelomycesContaminant
Ampelomyces
Non Ampelomyces
Outgroup
0.7 substitutions per site
